# Dental Instrument Exhibit at Panama-Pacific International Exposition Will Be of Educational Value to Public

**Published:** 1914-04-15

**Authors:** Waldemar H. F. N. Debille


### DENTAL INSTRUMENT EXHIBIT AT PANAMA-PACIFIC INTERNATIONAL EXPOSITION WILL BE OF EDUCATIONAL VALUE TO PUBLIC.

BY WALDEMAR H. F. N. DEBILLE.

To the average man, woman or child the dentist is the personification of his Satanic Majesty, and represents a survival of the torturer of the days of the Inquisition. That this view of the oldtime dentist was fully justified seems probable, and the general public is ignorant of the fact that today, with all the modern devices of science at its command, the dental profession is able to perform surgical and mechanical marvels without the patient being required to undergo any pain.

Every dentist realizes that it is a fact that the majority of persons will put off a trip to the dentist until the pain from a decayed tooth or exposed nerve is so great that of the two evils the surgeon's "torture" seems likely to prove the lesser. The natural result is that teeth which might have been saved are lost and pieces of porcelain must be substituted.

To offset this ignorance dental societies are attempting to educate the public by means of proper publicity. The ethical dentist is unable to carry on a campaign of education by individual advertising and must rely upon the group or association of which he is a part to bring the facts before the public. In a few notable instances this publicity has proved valuable, but in the majority of cities lack of co-operation has prevented the more modern dentists from accomplishing as much as should have been done.

It is doubtful if any one factor in the education of the public can compare with the comprehensive exhibits—which will be made of dental appliances and methods at the Panama-Pacific International Exposition. Several millions people from all parts of the world will visit this exposition and as it is a well-known psychological fact that one which is dreaded will attract the attention and interest, a large percentage of these visitors will make a careful inspection of the exhibits of dental instruments.

Without doubt there will be displayed every instrument now in use by the modern dentist and it is probable that these will be compared with the crude instruments of torture used by the dentists of even a decade ago.

A modern dental office will be shown in which will be displayed every kind of apparatus intended to alleviate and prevent pain and expert attendants will explain to the visiting public the reason why the modern Twentieth-century dentist is able to operate without fear of causing even the slightest temporary twinge of pain in the patient.

By education the people will soon learn to realize that the dread they have felt for dentists is inspired by non-existent cause — a true bugaboo — and thousands of teeth will be saved that would under present conditions of prevailing ignorance be entirely lost.

The exhibits at the Exposition should be elaborate and comprehensive. To ensure this, dental associations throughout the country should either co-operate to arrange for a combined exhibit, or the manufacturers of dental supplies should attend to the installation.

The exhibit is classified under the Department of Liberal Arts and will be displayed in the Palace of Liberal Arts, one of the handsomest buildings on the Exposition site.

